# Optimization of Control Point Layout for Orthophoto Generation of Indoor Murals

**DOI:** 10.3390/s25051588

**Published:** 2025-03-05

**Authors:** Dingfei Yan, Yongming Yang

**Affiliations:** 1 Faculty of Land and Resources Engineering, Kunming University of Science and Technology, Kunming 650031, China; yandingfei@stu.kust.edu.cn; 2 Faculty of Surveying and Information Engineering, West Yunnan University of Applied Sciences, Dali 671006, China; 3 Engineering Research Center for Spatial Atlas Information Protection of Ethnic Minority Murals and Rock Paintings, West Yunnan University of Applied Sciences, Dali 671006, China

**Keywords:** digital orthophoto, total station, RTK, control point deployment, spatial coordinate transformation, mural

## Abstract

This study focuses on the preservation of indoor murals, which can be supported by combining RTK and total station technology to explore the optimization of image geometric accuracy based on a control points layout. The study involves placing varying numbers of control points on the mural surface and processing the collected data using a spatial coordinate transformation model to assess the impact of different layouts on image accuracy. Some control points are used to ensure the spatial positioning accuracy of the images, while others serve as check points to validate the geometric precision of the images. After data processing, high-precision digital orthophotos are generated using Agisoft PhotoScan2.0.1 software, with accuracy verified by the check points. The experimental results show that as the number of control points increases, image accuracy improves gradually. When the number of control points reaches 24, the geometric accuracy of the images stabilizes, and further increases in the number of control points have a limited effect on improving accuracy. Therefore, the study proposes an optimal layout scheme: 24 control points for every 16 square meters. This scheme not only meets millimeter-level precision requirements but also effectively optimizes resource allocation and reduces time costs. The research provides reliable data support for the high-precision preservation and restoration of murals and offers important references for similar cultural heritage preservation projects.

## 1. Introduction

The digital orthophoto of the mural, as a key achievement in the digital preservation of murals, provides detailed and highly accurate high-resolution data, greatly advancing the long-term protection and restoration of the mural. Supported by photogrammetry technology, the digital orthophoto converts the mural’s three-dimensional surface information into a distortion-free two-dimensional image through precise image processing and geometric correction [1,2,3,4]. Compared to traditional flat photography, orthophotos eliminate geometric distortions caused by shooting angles and perspective, ensuring that the mural’s geometric form is accurately maintained. Traditional photography methods often fail to avoid image deformation due to angle variations or lens distortion, which can affect the faithful reproduction of the mural. In contrast, orthophotos correct these errors through geometric adjustments, offering higher spatial accuracy [5,6]. As a result, orthophotos not only more accurately reproduce the mural’s geometric shape but also provide reliable foundational data and reference value for subsequent detailed restoration, historical documentation, academic research, and virtual exhibitions. The application of this technology has significantly improved the accuracy and reliability of mural digital preservation, offering scientific technical support for cultural heritage conservation [7,8,9].

During the creation of digital orthophotos, the placement of control points is a critical factor in ensuring the geometric accuracy of the image [10]. Control points, which are reference points with known locations in space, primarily serve to establish an accurate geometric relationship between the image data and the real-world spatial coordinates. By strategically placing control points, consistency between the image and the physical space can be achieved, ensuring that each pixel in the generated digital orthophoto corresponds to its actual physical location in the scene, thus effectively maintaining the image’s scale and positional accuracy. The quantity, location, and spatial distribution of control points directly influence the geometric consistency and spatial precision of the image. A well-planned layout of control points ensures consistent accuracy across different areas of the image, reduces distortion and geometric errors, and improves image quality [11]. In the process of mural digitization, the proper placement of control points enables more precise reproduction of the mural, ensuring that no deformation or misalignment occurs during the digitization process, thereby more accurately reflecting the mural’s true appearance. Additionally, the optimized arrangement of control points can significantly reduce data collection costs. With the same level of accuracy requirements, a well-designed placement of fewer control points can drastically cut down on measurement and data collection time, making the process more efficient.

This study focuses on the digital preservation of indoor murals in temples, proposing an optimized control point layout strategy by combining RTK (Real-Time Kinematic) and total station technology [12]. The aim is to provide a reference for control point collection when creating similar orthophotos for other murals. Compared to traditional 3D laser scanning and SLAM technologies, the RTK and total station combination proposed in this study offers significant advantages in terms of cost and operational flexibility, particularly for cultural heritage preservation projects with limited resources [13,14,15,16]. By placing different numbers of high-precision control points, the study generates digital orthophotos and evaluates the geometric accuracy of the images using check points.

The experimental results demonstrate that both the number and the placement of control points directly affect image accuracy. An excessive number of control points not only increases measurement costs and time but may also result in resource waste. Conversely, too few control points may lead to a decrease in image accuracy. By analyzing errors with different control point layouts, the study ultimately identifies the optimal layout that balances precision, time, and labor costs. This layout provides a scientific reference for control point collection in other murals and allows for flexible adjustment of control point density and distribution strategies based on mural size and geometric characteristics.

Furthermore, this study establishes a local coordinate system linked to the CGCS2000 coordinate system, ensuring compatibility with other geographic information systems and facilitating subsequent data integration and long-term monitoring. This method not only improves data reusability but also provides a unified reference for cross-project comparisons and long-term preservation efforts. By linking the local coordinate system to the CGCS2000 system, the study presents an efficient and reliable technical solution for generating orthophotos of indoor murals with broad application prospects. This method not only meets the high-precision requirements for cultural heritage protection and restoration but also offers strong adaptability, making it applicable to other types of murals, cave murals, and outdoor rock art. It only requires adjustments to control point density and distribution strategies based on specific needs.

## 2. Principles and Methods

### 2.1. Principles of Control Point Deployment

In the production of digital orthophotos for murals, the proper deployment of control points is critical to ensuring geometric accuracy. To optimize the control point layout and enhance the geometric precision of the orthophotos, the following principles were adopted in this study:

(1) Uniform Distribution Principle

Control points should be as evenly distributed as possible across the mural area to minimize geometric distortions in different parts of the image. Sufficient control points should be allocated, particularly in the edge and central regions, to achieve balanced coverage and maintain overall geometric accuracy.

(2) Edge Priority Principle

The edges of murals are the most prone to geometric distortion. Therefore, priority is given to placing control points around the perimeter of the mural. This principle helps reduce edge distortions and ensures that geometric accuracy is evenly distributed throughout the image.

(3) Visibility and Angle Principle

The placement of control points should ensure that they remain unobstructed during image acquisition, allowing the equipment to accurately capture their data from multiple angles. In scenarios with limited space or poor lighting conditions, careful planning of angles and perspectives can effectively improve the quality of data collection.

(4) Precision Assurance Principle

The number of control points should be minimized while still meeting the required accuracy standards to reduce data collection time and costs. This principle ensures a balance between precision and efficiency.

### 2.2. Experimental Methods

The primary objective of this experiment is to generate digital orthophotos of murals by setting up varying numbers of control points, in order to explore the optimal control point layout and ensure the geometric accuracy of the generated images. One of the innovations of this study is that it provides a reference for collecting control points when creating similar orthophotos for other murals. Through a well-designed control point layout strategy and data processing workflow, this research offers a replicable process for the digital preservation of murals. The control point layout scheme in this study follows the 3 × 3 rules for photogrammetric documentation proposed by the International Committee for Documentation of Cultural Heritage (CIPA, 2013) [17]. These rules recommend (1) each image should contain at least 3 control points; (2) each control point should appear in at least 3 images; and (3) control points should be evenly distributed in three-dimensional space, covering different heights and depths of the subject. This rule ensures the robustness of geometric calibration during the photogrammetric process, while providing a reliable geometric foundation for subsequent orthophoto generation.

Due to the mural’s indoor location, traditional RTK surveying methods face significant limitations in this environment. Therefore, the experiment employed a combination of a prism-free total station and baseline measurement techniques to obtain the three-dimensional coordinates of the control points. The specific process is as follows: First, RTK technology was used to establish benchmark points outdoors, serving as the initial reference points for the measurements. Next, through a step-by-step introduction of the baseline measurement method, the total station equipment was moved indoors, ensuring precise measurement and collection of the three-dimensional coordinates for each control point located on the mural’s surface.

After data collection, all obtained control point data are input into a spatial coordinate transformation model for processing. The goal is to convert the raw three-dimensional coordinates into the target coordinate system required by Agisoft PhotoScan software [18]. Since the mural is located on a wall, the three-dimensional coordinate system of the control points collected on-site differs from the coordinate system required by Agisoft PhotoScan. Therefore, coordinate system conversion must be performed during the data processing stage. This conversion step is crucial as it ensures that the collected data align with the coordinate system needed by the image processing software, thereby guaranteeing the geometric accuracy of the final images.

After the conversion of control point coordinates, they were first categorized into two groups: one set was designated as control points, used to design various control point layout schemes. These control points provide precise spatial positioning data for subsequent conservation and restoration efforts, ensuring that these tasks are carried out on a reliable spatial foundation. The other set was designated as check points, used to validate the accuracy and precision of the images generated under different layout schemes. Based on the fundamental principles of control point layout, nine different schemes involving varying numbers of control points were designed, with control points distributed rationally across the surface of the mural according to these principles. The aim of these design schemes was to optimize the distribution of control points to enhance the geometric accuracy of the images, ensuring that the generated digital images exhibit spatial consistency and high precision, regardless of the number of control points used.

After designing the various control point layout schemes, the control points from each scheme, along with the collected image data, were imported into Agisoft PhotoScan software. The data were processed following a consistent image processing workflow, including aerial triangulation, geometric correction, point cloud densification, and texture mapping, ultimately resulting in the generation of digital orthophotos. Each layout scheme followed the same processing steps to ensure the scientific validity and comparability of the experimental results.

The second innovation of this study is the integration of a local coordinate system linked to the CGCS2000 coordinate system, which facilitates the integration of future cultural heritage data. This approach ensures data consistency and provides an efficient method for data alignment in subsequent cultural heritage preservation projects. By combining the local coordinate system with the national coordinate system, this method guarantees that the collected data can seamlessly integrate and be compared with other relevant data both nationally and internationally.

**Figure 1:**
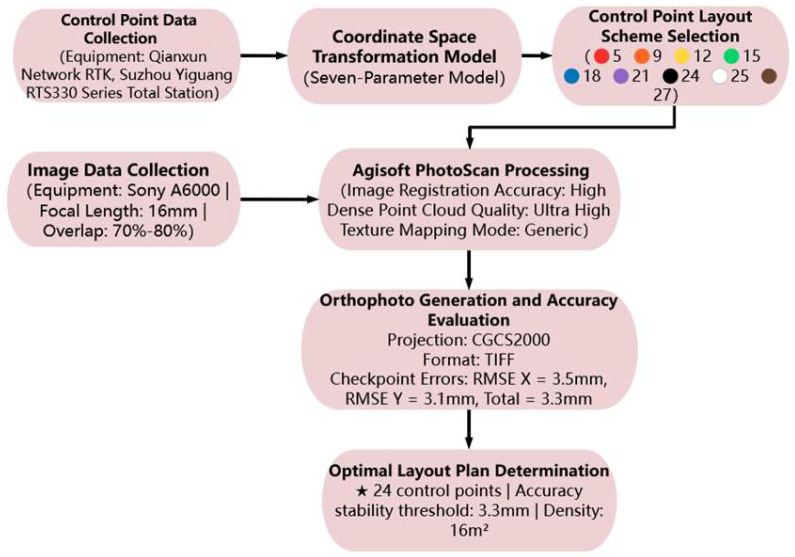
Experimental workflow.

Finally, the generated orthophotos from each layout scheme were subjected to error analysis using the check points. The errors at each check point, along with the overall error, were calculated to assess the accuracy of the images. Based on the results of the error analysis, the optimal control point layout scheme was selected. The overall experimental process is shown in Figure 1.

## 3. Study Area Overview

The study area is located in Lingxiang Temple, Xiaguan Town, Dali, Yunnan Province, China. The mural, situated within the temple, is a 4 m × 4 m indoor artwork with slightly concave edges on both sides. Due to the enclosed indoor environment, traditional RTK technology was unsuitable for the direct deployment and collection of control points. To address this limitation, this study employed traverse measurement techniques combined with a reflectorless total station to achieve high-precision collection of control point data. This method provides essential data support and technical assurance for optimizing the control point layout in the production of high-precision orthophotos for indoor murals.

## 4. Data Collection

### 4.1. Image Data Collection

In this study, image acquisition was conducted to provide the foundational data for generating digital orthophotos under different control point layout schemes, enabling the evaluation of the impact of control point deployment on orthophoto accuracy. To ensure data completeness and precision, the image acquisition process followed strict protocols, utilizing a Sony A6000 digital camera equipped with an SELP1650 lens to capture high-resolution mural images. Considering the unique conditions of the mural, such as its indoor location, insufficient lighting, and elevated height, a range of auxiliary equipment was employed, including a tripod, laser pointer, supplemental lighting, measuring tape, and ruler, to ensure stability and precision during image collection.

The acquisition process adhered to a predefined shooting route to guarantee uniform image coverage, with an overlap rate between adjacent images maintained at 70–80% to meet the requirements of subsequent image stitching and geometric correction. During the process, shooting parameters and image quality were continuously monitored and verified to ensure the collected data met the experimental requirements for precision and consistency. A total of 272 images were acquired, providing a robust dataset to support the optimization of control point deployment.

The image acquisition phase was designed with a focus on supporting control point optimization. By combining a scientifically planned acquisition process with high-precision equipment, this study provided reliable data, ensuring the academic rigor and scientific validity of the subsequent orthophoto generation and accuracy analysis. Detailed equipment parameters and camera specifications are shown in Table 1 and Table 2.

### 4.2. Control Point Data Collection

As the mural is located indoors, with control points placed in clearly identifiable areas, traditional RTK technology could not be directly applied to meet the measurement requirements. To address this limitation, this study employed a combined approach utilizing Qianxun Network RTK and the Suzhou Yiguang RTS330 series total station, ensuring the high-precision acquisition of control point coordinates. First, reference points were established outdoors using RTK technology, providing a benchmark for subsequent measurements. Next, traverse measurements were conducted with the total station, which was gradually moved into the indoor environment where the mural is located. Precise observations of the pre-defined control points were performed to obtain the three-dimensional coordinates of the mural’s control points, meeting the required experimental accuracy.

In the control point collection process, this study adopts a strategy combining ground control points and check points. The control points are used to provide absolute spatial positioning, ensuring an accurate mapping between the image data and the real-world space. In this study, control points are evenly distributed across the mural surface, with priority given to the edge areas to minimize geometric distortion and ensure the overall geometric accuracy of the image. The check points are used to independently verify the model’s accuracy, preventing overfitting. The locations of the check points are separate from those of the control points to ensure an objective assessment of the image’s geometric accuracy. Through this strategy, the study not only guarantees the overall accuracy of the image but also ensures the model’s reliability and robustness through independent verification with the check points.

Given the dim lighting and elevated position of the mural in the confined indoor space, the experiment optimized observation positions and equipment operations to ensure the total station’s proper functioning and improve the quality of control point acquisition. The total station was raised to its maximum height, and its position and viewing angles were carefully adjusted to optimize the observation path for each control point, ensuring that all points could be clearly observed. Additionally, a tripod equipped with supplemental lighting was used to enhance visibility under poor lighting conditions, further improving the clarity and accuracy of control point measurements.

In this study, during the total station traverse measurements, only one station was set up, so the impact of any measurement errors on the final results was negligible. Therefore, any minor errors that may have occurred during the traverse measurement process did not significantly affect the high-precision collection of control points.

**Figure 2:**
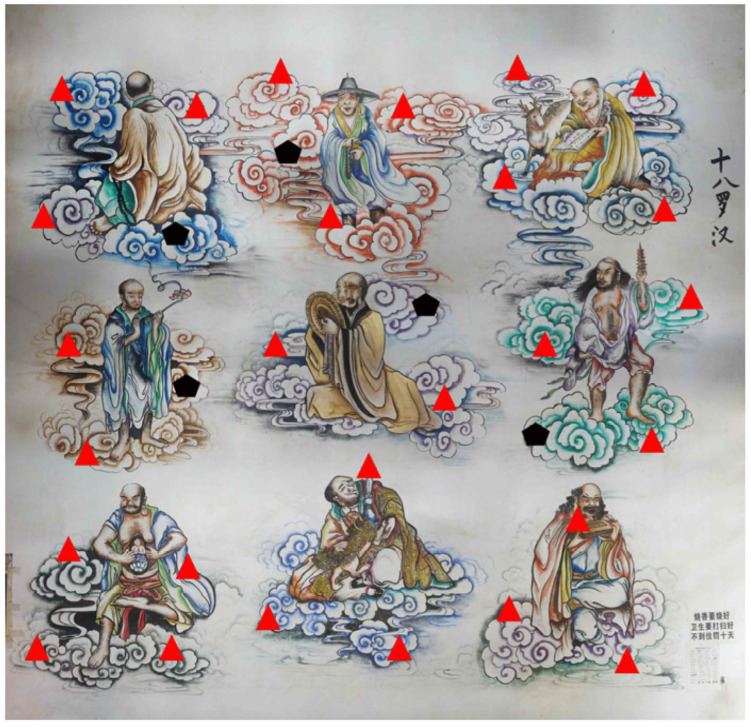
Control point distribution map. Note: Black pentagons represent the check points, and red triangles represent the control points.

By combining the strengths of RTK and reflectorless total station technologies, this experiment achieved efficient and precise acquisition of control point coordinates. This approach effectively overcame the challenges posed by the complex indoor environment, providing a robust foundation for subsequent image processing and the generation of digital orthophotos. The parameters for the RTK system and total station, as well as the distribution of collected control points, are detailed in Table 3, Table 4 and Table 5 and shown in Figure 2. This method not only ensures high-precision data acquisition but also serves as a valuable technical reference for the digital measurement of similar indoor cultural heritage artifacts.

As shown in the figure, a total of 32 control points were collected. The black pentagons represent the check points, which are used to assess image accuracy and verify the coordinate transformation model’s precision. The remaining red triangles represent the control points, which are involved in image processing. The placement of the check points plays a crucial role in validating the effectiveness of the control point layout and optimizing the geometric accuracy of the images, providing essential support for the scientific validity and reliability of the experimental results.

## 5. Control Point Layout Schemes

To optimize the control point layout for generating digital orthophotos of murals, this study designed nine layout schemes with varying numbers of control points, ensuring their reasonable distribution on the mural surface based on fundamental control point deployment principles [19,20,21,22,23,24,25]. As the mural is a valuable cultural artifact, direct contact or marking is strictly prohibited. To prevent any damage, control points were strategically placed on clearly visible and easily identifiable areas of the mural surface. A total of 32 control points were collected, of which five were designated as check points, while the remaining 27 were utilized in different layout schemes for image processing. This approach not only met the precision requirements for data collection but also ensured the non-destructive preservation of the artifact.

**Figure 3:**
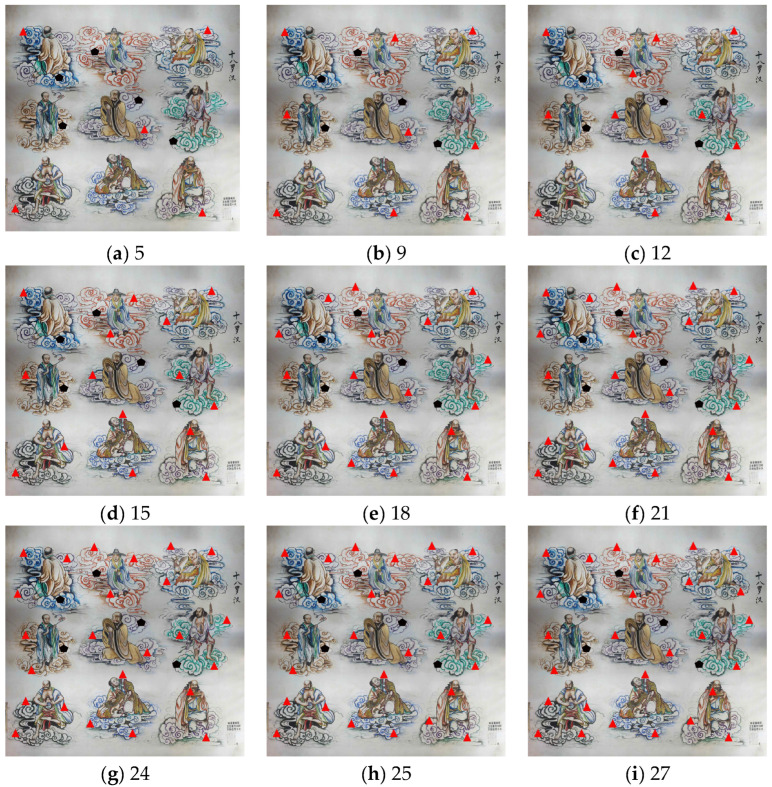
Schematic diagram of control point layout schemes. Note: Black pentagons represent the check points, and red triangles represent the control points.

For each layout scheme, the control points, along with the collected image data, were imported into Agisoft PhotoScan to generate digital orthophotos. The geometric accuracy of the resulting orthophotos was then evaluated. During the experiment, the number of control points was gradually increased (5, 9, 12, 15, 18, 21, 24, 25, and 27 points) to progressively improve the geometric accuracy of the orthophotos, meeting higher precision requirements and enabling more detailed image control. The distribution of control points for each layout scheme is shown in Figure 3.

As shown in Figure 3, the control point layout design follows the basic principles of control point distribution in low-altitude photogrammetry, while considering the specific characteristics of the mural. In scheme 1, the control points are placed at the four corners and the center of the mural to meet the basic geometric accuracy requirements. Schemes 2 through 9 gradually increase the number of control points based on scheme 1 to achieve higher accuracy. The numbers of control points in these schemes are 5, 9, 12, 15, 18, 21, 24, 25, and 27, progressively enhancing the image control capacity and geometric precision. All schemes adhere to the principles of uniform distribution and edge-priority placement. Control points are evenly distributed across the mural surface to ensure the overall geometric accuracy of the image. Additionally, control points are prioritized at the edges to reduce geometric distortion and ensure consistent accuracy in the edge regions. Through this strategy, the study is able to achieve high-precision image generation with varying numbers of control points.

## 6. Data Processing

### 6.1. Coordinate Space Transformation

Since the mural is located on the wall, the three-dimensional coordinate system of the collected control points differs from the specific coordinate system required by the image processing software, Agisoft PhotoScan. Directly using these raw control points for image processing would result in significant geometric errors in the generated digital orthophotos [26,27,28]. To ensure the accuracy and consistency of the image, this study employs a local coordinate system linked to the CGCS2000 coordinate system. Through a spatial coordinate transformation model, the original three-dimensional coordinate system is mapped to the specific coordinate system required by Agisoft PhotoScan. The transformed coordinates, using a seven-parameter coordinate transformation model, remain closely linked to CGCS2000, ensuring compatibility with other geographic information systems. This connection not only eliminates the deviation between the original data and the target system but also provides a unified reference for subsequent data integration and long-term monitoring.

This transformation process is a critical step in the creation of digital orthophotos, directly influencing the geometric accuracy and final application effectiveness of the images. Through precise coordinate mapping, this study provides an efficient and reliable technical solution for the digital preservation of indoor murals.

The mathematical expression of the seven-parameter model is as follows:

(1) $$\left[\begin{array}{l} X\\ Y\\ Z \end{array}\right]=\left[\begin{array}{l} T_{X}\\ T_{Y}\\ T_{Z} \end{array}\right]+\left(1+m\right)R_{3}\left(\omega_{X}\right)R_{2}\left(\omega_{Y}\right)R_{1}\left(\omega_{Z}\right)\left[\begin{array}{l} X_{0}\\ Y_{0}\\ Z_{0} \end{array}\right]$$

In the equation, (X_0_, Y_0_, Z_0_) represents the original coordinates of the control points before transformation, while (X, Y, Z) denotes the transformed coordinates. m is the scale factor, and (T_X_, T_Y_, T_Z_) are the translation parameters. ω_X_, ω_Y_, and ω_Z_ are the rotation angles around the X-axis, Y-axis, and Z-axis, respectively.

In Equation (1), the rotation matrices R_1_(ω_Z_), R_2_(ω_Y_), and R_3_(ω_X_) correspond to the rotations around the Z-axis, Y-axis, and X-axis, respectively. These are expressed as follows:

(2) $$\left\{\begin{array}{l} R_{1}\left(\omega_{Z}\right)=\left[\begin{array}{ccc} \cos\omega_{Z} & \sin\omega_{z} & 0\\ -\sin\omega_{z} & \cos\omega_{Z} & 0\\ 0 & 0 & 1 \end{array}\right]\\ R_{2}\left(\omega_{Y}\right)=\left[\begin{array}{ccc} \cos\omega_{Y} & 0 & -\sin\omega_{Y}\\ 0 & 1 & 0\\ \sin\omega_{Y} & 0 & \cos\omega_{Y} \end{array}\right]\\ R_{3}\left(\omega_{X}\right)=\left[\begin{array}{ccc} 1 & 0 & 0\\ 0 & \cos\omega_{X} & \sin\omega_{X}\\ 0 & -\sin\omega_{X} & \cos\omega_{X} \end{array}\right] \end{array}\right.$$

#### 6.1.1. Rotation Around the Y-Axis

**Figure 4:**
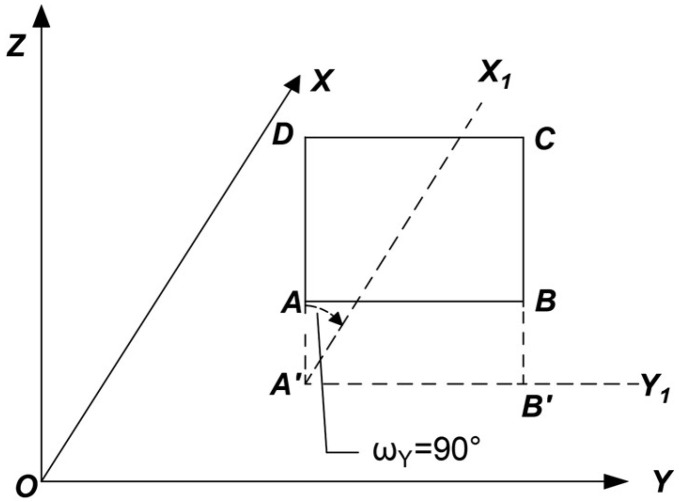
Before Y-axis rotation.

**Figure 5:**
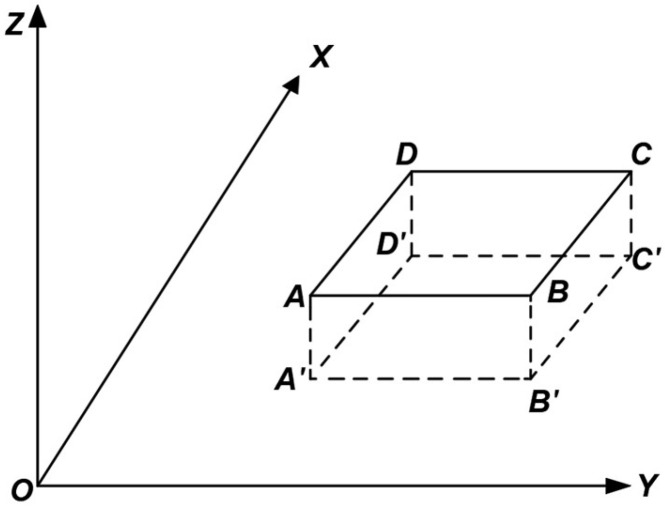
After Y-axis rotation.

As shown in Figure 4, the mural surface is assumed to be the fitted plane ABCD, which is perpendicular to the XOY plane. Its projection A’B’ on the XOY plane is parallel to the Y-axis. To meet the requirements of the coordinate system used by the image processing software, the plane ABCD needs to undergo a coordinate transformation by rotating 90° clockwise around the Y-axis. This adjustment aligns the plane with the software’s coordinate system. The result of the rotation is shown in Figure 5, where A’B’C’D’ represents the rotated projection of plane ABCD on the XOY plane.

This rotation process not only modifies the spatial positional relationship of the plane but also ensures consistency between the coordinate systems. This alignment is critical for subsequent geometric corrections and the generation of orthophotos, providing a solid foundation for achieving high-precision results.

As shown in Figure 4 and Figure 5, this experiment involves rotation only around the Y-axis and does not involve image scaling. Thus, by setting m = 0, ω_Z_ = 0°, and ω_X_ = 0°, and substituting these values into Equations (1) and (2), the rotational spatial model for this experiment can be expressed as

(3) $$\left[\begin{array}{c} X\\ Y\\ Z \end{array}\right]=\left[\begin{array}{c} T_{X}\\ T_{Y}\\ T_{Z} \end{array}\right]+\left[\begin{array}{ccc} \cos\omega_{Y} & 0 & -\sin\omega_{Y}\\ 0 & 1 & 0\\ \sin\omega_{Y} & 0 & \cos\omega_{Y} \end{array}\right]\left[\begin{array}{c} X_{0}\\ Y_{0}\\ Z_{0} \end{array}\right]$$

#### 6.1.2. Translation Parameters

After the aforementioned rotation process, the coordinates of the mural control points underwent significant changes, resulting in misalignment with the specific coordinate system required by the image processing software. As a result, these transformed coordinates cannot be directly applied to image processing. To address this issue, it is necessary to introduce appropriate translation parameters to correct the coordinate shifts caused by the rotation process [29,30]. The formula for calculating the translation parameters is as follows:

(4) $$\left\{\begin{array}{c} T_{X}=\sum_{i=1}^{n}X_{0}^{i}/n-\sum_{i=1}^{n}X^{i}/n\\ T_{Y}=\sum_{i=1}^{n}Y_{0}^{i}/n-\sum_{i=1}^{n}Y^{i}/n\;\\ T_{Z}=\sum_{i=1}^{n}Z_{0}^{i}/n-\sum_{i=1}^{n}Z^{i}/n \end{array}\right.$$

In the formula, nn represents the number of control points, X^i^, Y^i^, Z^i^ are the coordinates obtained after the rotation, and $X_{0}^{i}$, $Y_{0}^{i}$, $Z_{0}^{i}$ are the coordinates of the control points before the rotation.

#### 6.1.3. Coordinate Transformation Model

In this experiment, a total of 32 control points were collected, with the mural’s fitted plane ABCD assumed to be parallel to the XOY plane and perpendicular to the Y-axis. To achieve the coordinate system transformation, it is only necessary to rotate this plane 90° clockwise around the Y-axis. According to the right-hand rule of the coordinate system, the rotation angle is set to −90°. By substituting this angle into Equation (3), the specific calculation results are as follows:

(5) $$\left[\begin{array}{c} X\\ Y\\ Z \end{array}\right]=\left[\begin{array}{c} T_{X}\\ T_{Y}\\ T_{Z} \end{array}\right]+\left[\begin{array}{ccc} 0 & 0 & 1\\ 0 & 1 & 0\\ -1 & 0 & 0 \end{array}\right]\left[\begin{array}{c} X_{0}\\ Y_{0}\\ Z_{0} \end{array}\right]$$

To determine the translation parameters, the initial translation parameters are set to zero, and the coordinates X^i^, Y^i^, Z^i^ of the control points are calculated based solely on rotation. These values are then substituted into Equation (4) to derive the translation parameters for the coordinate transformation, which are expressed as follows:

(6) $$\left[\begin{array}{c} T_{X}\\ T_{Y}\\ T_{Z} \end{array}\right]=\left[\begin{array}{c} 2835597.8762\\ 1243284.0250\\ 2835597.8762 \end{array}\right]$$

Substituting Equation (6) into Equation (5), the final spatial transformation model is obtained as follows:

(7) $$\left[\begin{array}{c} X\\ Y\\ Z \end{array}\right]=\left[\begin{array}{c} 2835597.8762\\ 1243284.0250\\ 2835597.8762 \end{array}\right]+\left[\begin{array}{ccc} 0 & 0 & 1\\ 0 & 1 & 0\\ -1 & 0 & 0 \end{array}\right]\left[\begin{array}{c} X_{0}\\ Y_{0}\\ Z_{0} \end{array}\right]$$

By substituting the coordinate values from Table 5 into Equation (7), the transformed coordinates of the control points for this experiment are obtained, as shown in Table 6.

The coordinates of the check points are as follows in Table 7:

### 6.2. Orthophoto Generation

**Figure 6:**
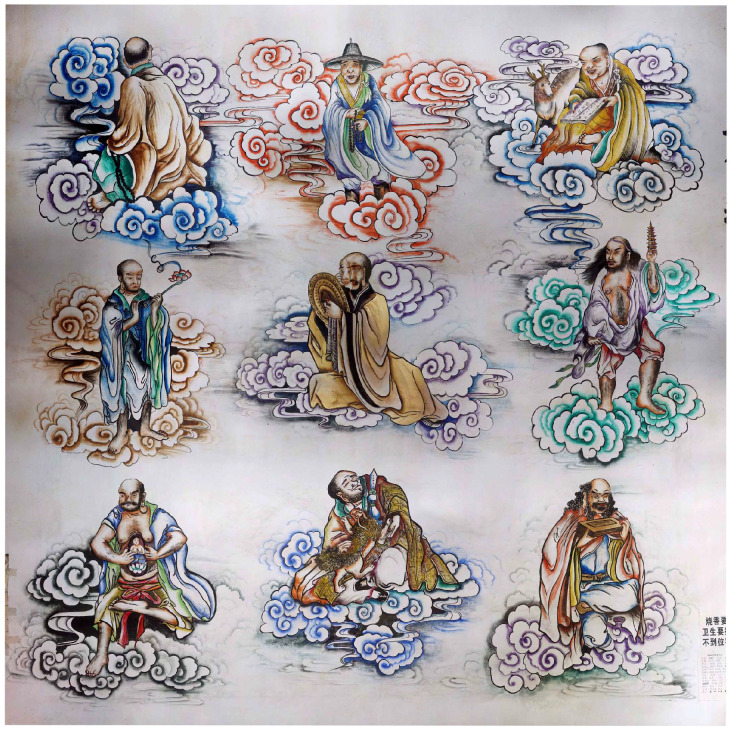
Digital orthophoto generated with 24 control points.

Based on the designed control point distribution schemes with varying numbers of control points, this experiment imported the transformed coordinates of the control points, along with the corresponding image data, into the Agisoft PhotoScan software for processing [31,32,33]. Within the software, digital orthophotos were generated through geometric constraints applied to the control points, ensuring alignment between the image and the control point coordinates to meet the desired geometric accuracy requirements. The image processing for each distribution scheme strictly followed a standardized workflow to ensure consistency and comparability. This approach effectively enabled a detailed comparison of the geometric accuracy across different schemes, providing reliable experimental data to identify the optimal control point distribution. The final generated example of the orthophoto is shown in Figure 6.

## 7. Accuracy Analysis

To determine the optimal control point layout, this study imported orthophotos generated from different layout schemes into image measurement software, using the established check points for coordinate measurement. The measured coordinates were then compared to the actual values, which were transformed into the required coordinate system, and the results were calculated using Equation (8). The calculation results are shown in Table 8. Based on the deviation values, the root mean square error (RMSE) for the check points in each scheme was further calculated, along with the total RMSE, to evaluate the accuracy performance of different layout schemes. Specifically, for each layout scheme, the RMSE in both the X and Y directions was calculated independently to assess the geometric accuracy in each direction. Then, the total RMSE formula was used to combine the RMSE in the X and Y directions into an overall error metric.

Additionally, this study systematically evaluated the distribution of control points. All schemes followed the principles of uniform distribution and edge-priority placement: control points were evenly distributed across the mural surface to ensure overall geometric accuracy, with priority given to the edges to minimize geometric distortion. Experimental results show that the edge-priority placement strategy significantly reduced errors in the edge regions, while the uniform distribution ensured consistency in precision across different areas of the image.

This method effectively compares the impact of different control point numbers and layout strategies on image accuracy. The formulas for calculating deviation, root mean square error, and total root mean square error are as follows [34,35].

(8) $$E=x_{i}-\hat{x}$$

(9) $$M=\sqrt{\frac{1}{N}\sum_{i=1}^{N}{\left(x_{i}-{\hat{x}}_{i}\right)}^{2}}$$

(10) $$W=\sqrt{\frac{M_{X}^{2}+M_{Y}^{2}}{2}}$$

where E represents the deviation between the measured value and the actual value, x is the actual value, ${\hat{x}}_{i}$ is the measured value, M is the root mean square error, N is the number of check points, and W is the total root mean square error.

**Figure 7:**
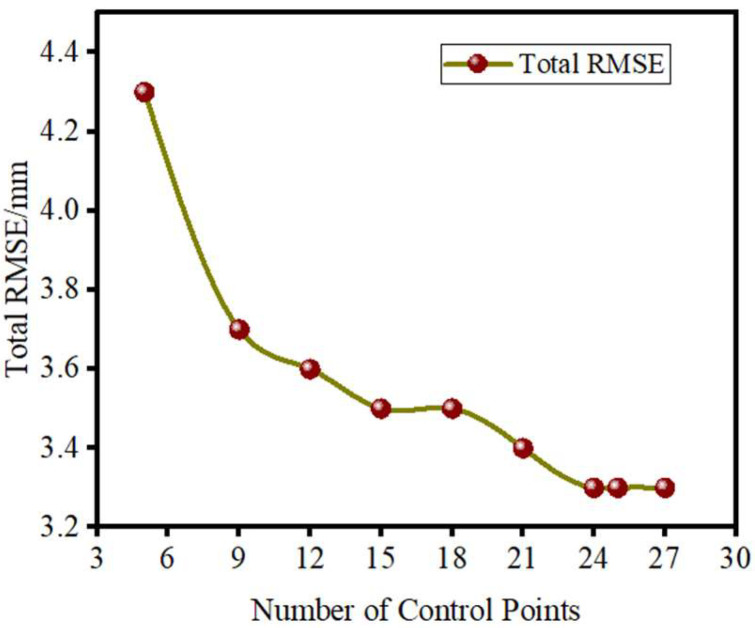
Total root mean square error distribution.

During the experiment, as the number of control points increased, the RMSE of the check points and the total RMSE progressively decreased, showing a clear trend of improved accuracy. By analyzing the variation in total RMSE, it was determined that the optimal number of control points is reached when the RMSE stabilizes at a smaller value. This layout not only ensures that the geometric accuracy of the image meets the required standards, but also effectively reduces the cost of control point setup and data collection. It strikes an optimal balance between image accuracy and resource investment. Based on the nine different control point layout schemes, the data from Table 8 were input into Formulas (9) and (10) for calculation, and the results were analyzed for accuracy. The analysis results are presented in Table 9 and Figure 7.

This experiment gradually increased the number of control points to generate digital orthophotos and conducted error analysis on the accuracy of different layout schemes. The results indicate that as the number of control points increases, the geometric accuracy of the image significantly improves, with the total root mean square error (total RMSE) decreasing from 4.3 mm in the initial scheme to 3.3 mm, greatly enhancing the overall accuracy. However, when the number of control points reached 24, the total error stabilized, and further increases in control points had a minimal effect on accuracy, indicating that this layout scheme reached its optimization limit in terms of precision.

It is important to note that the mural wall has a slight concave structure, which affects the error distribution differently. The concave structure has a more significant impact on the X-axis, with the error in the X-direction continuing to decrease as the number of control points increases. In contrast, the error in the Y-direction stabilized after 15 control points. Despite this, the trend in Total RMSE suggests that the increase in control points still has a significant effect on improving overall accuracy.

To further analyze the time cost and efficiency of the optimal control point layout scheme, this study compared the data collection time, data processing time, and Total RMSE for different numbers of control points. The specific data are shown in Table 10.

From Table 10, it can be seen that as the number of control points increases, the total root mean square error (Total RMSE) gradually decreases. When the number of control points reaches 24, the total error stabilizes, and further increases in control points have limited impact on improving accuracy. At the same time, increasing the number of control points significantly increases the time and cost of data collection and processing. For example, when the number of control points increased from 21 to 27, the data collection time increased by 12.5%, but the accuracy only improved by 0.1 mm. Therefore, this study concludes that 24 control points is the optimal configuration, which ensures high accuracy while optimizing time and cost.

Based on the above analysis, further examination of the RMSE and Total RMSE leads to the conclusion that when the number of control points is 24, the accuracy in the X-direction is optimized, while the Y-direction error stabilizes at 15 control points. With 24 control points, the Total RMSE remains stable at 3.3 mm, and both the X and Y directions reach high levels of geometric accuracy. This indicates that 24 control points is the optimal configuration for this experiment, ensuring that the image meets the high accuracy requirements while maintaining high efficiency in data collection and processing, and avoiding the time and cost wastage caused by excessive control points. Thus, this study establishes that 24 control points is the optimal layout for control points, ensuring both precision and cost-effectiveness, and provides an effective strategy for the reasonable placement of control points in cultural heritage preservation.

## 8. Conclusions

This study focuses on the digital preservation of the 4 m × 4 m mural at Lingxiang Temple and proposes an innovative control point layout plan that addresses the limitations of traditional RTK technology in narrow, poorly lit indoor environments. The study employs a combination of low-altitude photogrammetry and total station methods, utilizing a spatial transformation model to generate high-precision digital orthophotos. By strategically placing control points, the study ensures the spatial accuracy of the images, providing reliable data for subsequent cultural heritage preservation and restoration efforts.

Experimental results indicate that as the number of control points increases, the geometric accuracy of the images gradually improves. However, when the number of control points reaches 24, the accuracy improvement begins to level off. Based on this, this study identifies 24 control points as the optimal configuration, which maximizes the optimization of time and cost while ensuring high precision. This provides an efficient solution for mural digital preservation.

The innovations in this study are twofold. First, it proposes a reproducible control point layout plan tailored for mural digital preservation, suitable for similar cultural heritage projects in comparable environments. Through well-designed control point placement and data processing workflows, the study provides a valuable technical reference for the digital preservation of other murals. Second, it incorporates a local coordinate system linked to CGCS2000, facilitating the integration of cultural heritage data in subsequent work. This method ensures data consistency and enables seamless integration with other cultural heritage datasets, fostering data sharing and cross-disciplinary collaboration. Moreover, this approach is not limited to mural preservation but can be extended to applications such as cave murals, outdoor rock art, and other cultural heritage scenarios. The key is to adjust the control point density proportionally based on the specific context to ensure effective application across different environments.

Compared to traditional three-dimensional laser scanning and SLAM technologies, the method proposed in this study offers advantages such as low cost, low energy consumption, and ease of operation, making it especially suitable for resource-constrained cultural heritage preservation projects. Additionally, the use of a local coordinate system related to CGCS2000 aids in subsequent data integration and sharing, promoting cross-regional cooperation in the field of cultural heritage preservation.

Nevertheless, this study does have certain limitations. First, the experiment was only validated at Lingxiang Temple, and future research could expand to other murals and indoor environments to assess its applicability. Second, low-altitude photogrammetry is highly influenced by lighting conditions, and future work could integrate other optical measurement methods to enhance accuracy. Finally, future studies could optimize control point placement strategies based on variations in mural surface details to further improve image precision.

## Figures and Tables

**Table 1:** Parameters of image acquisition equipment.

Equipment	Specifications
Camera	Sony A6000
Lens	SELP1650
Steel Tape Measure	10 m
Cloth Tape Measure	10 m
Tripod	1.78 m–6.1 m

**Table 2:** Camera parameters.

Imaging Parameters	Specifications
Focal Length	16 mm
Sensor Size	23.5 mm × 15.6 mm
Image Resolution	6000 × 4000 (pixels)
Pixel Size	0.00392 mm
Effective Shooting Distance	≥25 cm

**Table 3:** Parameters of the RTK system.

Parameter Name	Specification
Number of Channels	800
Horizontal Static Accuracy	±(2.5 mm + 0.5 × 10^−6^ × D)
Vertical Static Accuracy	±(5.0 mm + 0.5 × 10^−6^ × D)
Horizontal RTK Accuracy	±(5.0 mm + 0.5 × 10^−6^ × D)
Vertical RTK Accuracy	±(5.0 mm + 0.5 × 10^−6^ × D)
Initialization Time	10 s
Supported Coordinate Systems	WGS-84, CGCS2000, etc.

**Table 4:** Parameters of the total station.

Parameter Name	Specification
Minimum Observation Distance	1.0 m
Distance Measurement Accuracy	2 mm + 2 × 10^−6^ × D
Measurement Range	1000 m (reflectorless), 1200 m (reflective target), 5000 m (single prism)
Angular Measurement Accuracy	2″
Compensation Accuracy	1″

**Table 5:** Image control point coordinates.

Serial Number	X (m)	Y (m)	Z (m)
PT1	2,833,609.1640	621,640.9092	1990.2913
PT2	2,833,609.1650	621,640.1129	1989.5557
PT3	2,833,609.1650	621,640.8998	1989.4680
………	………	………	………
PT16	2,833,609.1600	621,643.9145	1988.1582
PT17	2,833,609.1590	621,643.1151	1988.2473
PT18	2,833,609.1610	621,643.1779	1988.7685
………	………	………	………
PT30	2,833,609.1690	621,640.2000	1990.3703
PT31	2,833,609.1640	621,641.5274	1990.4706
PT32	2,833,609.1600	621,640.8825	1986.9148

**Table 6:** Coordinates after image control point transformation.

Serial Number	X_1_ (m)	Y_1_ (m)	Z_1_ (m)
PT1	2,833,607.5849	621,643.1158	1988.7122
PT2	2,833,608.3205	621,643.9121	1988.7112
PT3	2,833,608.4082	621,643.1252	1988.7112
………	………	………	………
PT16	2,833609.7180	621,640.1105	1988.7162
PT17	2,833,,609.6289	621,640.9099	1988.7172
PT18	2,833,609.1077	621,640.8471	1988.7152
………	………	………	………
P30	2,833,607.5059	621,643.8250	1988.7072
P31	2,833,607.4056	621,642.4976	1988.7122
P32	2,833,610.9614	621,643.1425	1988.7162

**Table 7:** Coordinates of check points after transformation.

Serial Number	X_1_ (m)	Y_1_ (m)	Z_1_ (m)
PT3	2,833,608.4082	621,643.1252	1988.7112
PT5	2,833,609.4287	621,643.0194	1988.7162
PT17	2,833,609.6289	621,640.9099	1988.7172
PT26	2,833,607.8694	621,642.4366	1988.7112
PT27	2,833,608.7893	621,641.5604	1988.7172

**Table 8:** Differences between measured and actual values for different control point quantities.

Control Point Quantity/Check Point ID		3	5	17	26	27
5	EX	0.0036	−0.0036	0.0011	0.0080	−0.0016
	EY	0.0066	0.0033	0.0059	−0.0004	−0.0010
9	EX	0.0024	−0.0043	0.0005	0.0072	−0.0019
	EY	0.0057	0.0023	0.0037	−0.0005	−0.0019
12	EX	0.0020	−0.0045	0.0004	0.0068	−0.0021
	EY	0.0059	0.0025	0.0042	−0.0004	−0.0033
15	EX	0.0015	−0.0046	0.0001	0.0070	−0.0021
	EY	0.0054	0.0024	0.0034	0.0001	−0.0016
18	EX	0.0020	−0.0041	−0.0002	0.0069	−0.0018
	EY	0.0037	0.0020	0.0040	−0.0008	−0.0015
21	EX	0.0020	−0.0031	0.0005	0.0072	−0.0009
	EY	0.0052	0.0017	0.0043	0.0004	−0.0005
24	EX	0.0014	−0.0040	0.0016	0.0063	−0.0012
	EY	0.0051	0.0017	0.0042	0.0001	−0.0010
25	EX	0.0020	−0.0031	0.0007	0.0067	−0.0009
	EY	0.0047	0.0026	0.0041	−0.0005	−0.0011
27	EX	0.0017	−0.0034	0.0010	0.0066	−0.0009
	EY	0.0036	0.0028	0.0046	−0.0004	−0.0014

**Table 9:** Error results of different control point schemes.

Control Point Scheme	Number of Control Points	Number of Check Points	Check Point RMSE/mm		Total RMSE/mm
			ΔX	ΔY	
1	5	5	4.3	4.3	4.3
2	9	5	4.0	3.3	3.7
3	12	5	3.9	3.3	3.6
4	15	5	3.9	3.1	3.5
5	18	5	3.8	3.1	3.5
6	21	5	3.6	3.1	3.4
7	24	5	3.5	3.1	3.3
8	25	5	3.5	3.1	3.3
9	27	5	3.5	3.1	3.3

**Table 10:** Data collection time, data processing time, and total root mean square error for different numbers of control points.

Number of Control Points	Data Collection Time (Hours:Minutes)	Data Processing Time (Hours:Minutes)	Total RMSE (mm)
5	0:15	2:59	4.3
9	0:27	2:57	3.7
12	0:36	2:57	3.6
15	0:45	3:01	3.5
18	0:54	2:57	3.5
21	1:03	3:01	3.4
24	1:12	3:01	3.3
25	1:15	3:05	3.3
27	1:21	3:06	3.3

## Data Availability

The raw data supporting the conclusions of this article will be made available by the authors on request.
